# The role of epigenetics and miRNAs in neuroblastoma development

**DOI:** 10.1186/1753-6561-6-S4-O11

**Published:** 2012-07-09

**Authors:** KV Rupani, S Das, RL Stallings

**Affiliations:** 1 Department of Cancer Genetics, Royal College of Surgeons in Ireland; 2 Children's Research Center, Our Lady's Children's Hospital, Crumlin, Ireland

## Introduction

Neuroblastoma (NB) is an often fatal pediatric cancer that arises from the precursor cells of the sympathetic nervous system. 13-Cis retinoic acid is used to treat high-risk disease. A derivative, all-trans-retinoic acid (ATRA) causes genome-wide demethylation in NB cells by up-regulating miRNAs such as miR-152 and miR-26a/b which are predicted to target the methyltransferases *DNMT1* and *DNMT3b*, respectively. The purpose of this study was to test whether ectopic over-expression of miR-26a/b, a known tumor suppressor miRNA in several other cancer types, led to reduced cell viability and *DNMT3b* expression in SK-N-BE NB cells. In addition, we also carried out a methylated DNA immunoprecipitation to microarrays following miR-152 over-expression in SK-N-BE cells in order to assess whether the ectopic over expression of miR-152 had any effects on genome-wide methylation.

## Results

Results showed that reduced miR-26a/b expression correlates to poor survival in patients with NB. We investigated the possibility of epigenetic factors that could control miR-26a/b expression in NB cells by treating the cells with a DNA-demethylating agent (5'-Aza-2 deoxycytodine). We did not observe any significant re-expression of miR-26a/b after application of the drug. It was further shown that ectopic over-expression of miR-26a causes a down-regulation of DNMT3b mRNA. Cell viability assays carried out following ectopic over-expression of miR-26a supported its possible involvement in reducing cell growth levels 96 hours post-transfection. Using the SHEP-TET NB cell line system, a *MYCN* induced repression of miR-26a/b in NB was also ruled out. Genome-wide methylation analysis following over-expression of miR-152 revealed genes that display overlapping methylation patterns as seen with ATRA treatment alone, thus allowing the identification of genes that are possibly controlled by miR-152 following suppression of DNMT1.

## Conclusions

Having ruled out DNA methylation and MYCN as significant regulators of miR-26a/b expression during NB differentiation, we propose that other transcription factors and/or retinoic acid directly may regulate this microRNA. In addition we have identified several genes that are epigenetically regulated through the action of miR-152 and warrant further follow up to determine their importance in NB differentiation.

